# First‐line therapy for post‐traumatic stress disorder: A systematic review of cognitive behavioural therapy and psychodynamic approaches

**DOI:** 10.1002/capr.12174

**Published:** 2018-07-03

**Authors:** Emma Paintain, Simon Cassidy

**Affiliations:** ^1^ Psychology University of Salford Salford UK

**Keywords:** cognitive behaviour therapy, dropout, exposure, posttraumatic stress disorder, psychodynamic therapy, therapist drift

## Abstract

**Background:**

Despite evidence supporting cognitive behavioural therapy (CBT)‐based interventions as the most effective approach for treating post‐traumatic stress disorder (PTSD) in randomised control trials, alternative treatment interventions are often used in clinical practice. Psychodynamic (PDT)‐based interventions are one example of such preferred approaches, this is despite comparatively limited available evidence supporting their effectiveness for treating PTSD.

**Aims:**

Existing research exploring effective therapeutic interventions for PTSD includes trauma‐focused CBT involving exposure techniques. The present review sought to establish the treatment efficacy of CBT and PDT approaches and considers the potential impact of selecting PDT‐based techniques over CBT‐based techniques for the treatment of PTSD.

**Results:**

The evidence reviewed provided examples supporting PDT‐based therapy as an effective treatment for PTSD, but confirmed CBT as more effective in the treatment of this particular disorder. Comparable dropout rates were reported for both treatment approaches, suggesting that relative dropout rate should not be a pivotal factor in the selection of a PDT approach over CBT for treatment of PTSD.

**Conclusion/Implications:**

The need to routinely observe evidence‐based recommendations for effective treatment of PTSD is highlighted and factors undermining practitioner engagement with CBT‐based interventions for the treatment of PTSD are identified.

## Introduction

The present review explores an apparent incongruence between the use of cognitive behaviour therapy (CBT) as the recommended first‐line therapy for the treatment of post‐traumatic stress disorder (PTSD) (Kar, 2011; National Institute for Clinical Excellence, 2005), and the therapy suggested as more often used by clinicians in practice, psychodynamic therapy (PDT) (Cahill, Foa, Hembree, Marshall & Nacasch, 2006; Foa, Gillihan & Bryant, 2013). Evidence relating to the efficacy of both CBT and PDT is reviewed and factors identified as relevant to practitioners’ decisions regarding treatment approach are considered in the light of the evidence.

Post‐traumatic stress disorder is defined as a psychiatric sequel following an individual's exposure to a traumatic situation of a threatening or catastrophic nature (Kar, 2011). PTSD is classified according to the re‐experiencing of the traumatic event, avoidance of stimuli associated with the traumatic event, hyperarousal and hypervigilance (Benish, Imel & Wampold, 2008). The Diagnostic and Statistical Manual (DSM‐5) (American Psychiatric Association, 2013) now includes PTSD in the category of trauma and stress or related disorders. The diagnostic criteria presented in the DSM‐5 identifies exposure to actual or threatened death, serious injury or sexual violation as the required trigger for the disorder, with the individual directly experiencing or witnessing the traumatic event or learning that the traumatic event occurred to a close family member or close friend, or experiences, first‐hand, repeated or extreme exposure to aversive details of the traumatic event. The criteria stipulate that ‘The disturbance, regardless of its trigger, causes clinically significant distress or impairment in the individual's social interactions, capacity to work or other important areas of functioning’ (APA, 2013, para. 3).

According to Foa et al. (2013) and Javidi and Yadollahie (2012), the majority of the population, 60.7% of men and 51.2% of women, are exposed to a traumatic event at some point during their lifetime which has the potential to trigger the onset of PTSD. The incidence of PTSD is reported to be as high as 6.8% in the US, increasing in military personnel and veterans of war in Iraq and Afghanistan to 13.8% meeting DSM diagnostic criteria for PTSD (Kearns, Ressler, Zatzick & Rothbaum, 2012). PTSD tends to have a chronic course, with 40% of sufferers experiencing symptoms to a significant degree, years after initial onset (Kearns et al., 2012).

Diagnosis of PTSD is accompanied by direct and indirect public health consequences including suicide, secondary mental health disorders, substance abuse, impaired functioning, health problems and reduced life course opportunities (Roberts, Gilman, Breslau, Breslau & Koenen, 2011). Significant associations have also been reported between PTSD and depression, and between PTSD and self‐reported health problems (Possemato, Wade, Andersen & Ouimette, 2010). PTSD has been shown to have a severe impact on functioning, leading to an increase in the risk of unemployment by as much as 150%, marital instability by 60%, and suicide for sufferers exceeding that of any other anxiety disorder (Galovski & Lyons, 2004). The potential negative impact of PTSD for the individual, their family, and wider society highlights a clear imperative for diagnosis and effective treatment of the disorder.

Despite some evidence suggesting poor response rates and limited long‐term effects (Durham et al., 2005; Schottenbauer, Glass, Arnkoff, Tendick & Gray, 2008), CBT is recommended by expert panels and in treatment guidelines as the first‐line therapy for the treatment of PTSD (Kar, 2011; NICE, 2005). A meta‐analysis of randomised treatment studies for PTSD conducted by Bradley, Greene, Russ, Dutra and Westen (2005) identified 26 studies utilising 44 treatment conditions, of which 37 were classified as CBT and 23 as control conditions. This study reported a large mean effect size based on pretreatment to post‐treatment change across treatments of 1.43, with 67% of treatment completers no longer meeting the criteria for PTSD. Similar results are reported in more recent reviews by Ulrich, Tran and Gregor (2016), Ehring et al. (2014), and Hofmann, Asnaani, Vonk, Sawyer and Fang (2012).

Specifically, trauma‐focussed (TF) CBT methods incorporating exposure techniques seem the most effective and widely recommended treatment modalities (Cukor, Spitalnick, Difede, Rizzo & Rothbaum, 2009). Exposure to the memory of traumatic events is a common feature of evidence‐based interventions for PTSD and is considered to be a critical element of CBT techniques (Peri, Gofman, Tal & Tuval‐Mashiach, 2015). Following an expert panel review, The Institute of Medicine (2008) reported that exposure therapy is the only treatment with enough empirical evidence to support its recommendation for the treatment of PTSD.

While all TF‐CBT protocols involve the patient confronting trauma memories and reminders of the traumatic event, emphasis on the traumatic event and its method vary according to the particular model of exposure adopted (Ehlers, Clark, et al, 2010; Gaston, 2015). Prolonged exposure (PE) (Foa & Rothbaum, 1998) is a highly trauma‐focused technique involving the patient repeatedly reliving (imagined or in real life/in vivo) the traumatic event to provoke anxiety. Through habituation, PE seeks to desensitise the patient so that the traumatic event is no longer associated with feelings of anxiety (Foa & Meadows, 1997; Gaston, 2015). Cognitive processing therapy (CPT) (Resick & Schnicke, 1992), although still trauma‐focused, does not involve reliving the traumatic event as vividly as it is relived in PE. Instead, the patient writes a detailed narrative account of the traumatic event, experiencing the associated emotions in a bid to identify dysfunctional cognitions which will then become the focus of cognitive reprocessing and re‐structuring (Gaston, 2015). Ehlers, Clark, Hackmann, McManus and Fennell (2005) cognitive therapy for PTSD (CT‐PTSD) includes narrative writing to identify the most distressing elements of the traumatic event (hot spots). Thus, depending on the particular technique used, exposure may involve actively testing the individual's faulty cognitions through the use of behavioural experiments, repeated exposure to attempt to reduce the intensity of fear responses and eliminate avoidance responses, and improve skills for handling feared situations (Gunter & Whittal, 2010).

The literature also supports alternative forms of TF‐CBT, including eye movement desensitisation and reprocessing (EMDR) and cognitive processing therapy (CPT) as effective treatments for PTSD (Hofmann et al., 2012; Holliday, Link‐Malcolm, Morris & Surı′s, 2014; National Institute for Clinical Excellence, 2005; Ulrich et al., 2016). Nevertheless, in a review exploring natural disasters, man‐made traumas, sexual abuse, refugee samples, disaster workers, terrorism, road traffic accidents, and war trauma, Kar (2011) reported CBT as an effective form of treatment for both acute and chronic PTSD, producing both short‐term and long‐term benefits and with preventative potential for PTSD – subject to further evidence. Despite the substantial support for CBT, there is evidence that mental health practitioners do not use CBT treatments consistently and most patients suffering from PTSD are not treated using CBT techniques (Cahill et al., 2006; Foa et al., 2013; Kar, 2011; Olatunji, Deacon & Abramowitz, 2009). In a study of primary care patients, similar numbers of patients were found to have received CBT (30%) and PDT (32%) (Schottenbauer et al., 2008).

Suggesting reasons why CBT may be underutilised by practitioners, van Minnen, Hendriks and Olff (2010) report lack of training and experience, perceived credibility, fear of increasing distress or exacerbating symptoms as pertinent factors. Cahill et al. (2006) and Follette and Ruzek (2007) also highlight concerns about the safety of exposure therapy, with exposure elements of CBT causing further emotional distress (Gaston, 2015). In support of the view that PTSD sufferers may not have the capacity to cope with trauma‐focused therapies and are thus hesitant when using these techniques with PTSD clients, Becker, Zayfert and Anderson (2004) found that only 4% of therapists reported using prolonged exposure in their treatment of PTSD and viewed potential treatment side effects as a barrier to its implementation.

Treatments incorporating exposure can re‐expose patients to the traumatic event and discourage them from continuing treatment and psychotherapies viewed as harmful may return high rates of premature treatment termination (dropout), representing a potential barrier to implementation (Bleiberg & Markowitz, 2005; Follette & Ruzek, 2007; Lilienfeld, 2007). In support of this suggestion, completion rates for CBT in clinical settings tend to be markedly lower than those reported in randomised control trials (Hans & Hiller, 2013; Kar, 2011; Zayfert et al., 2005). McDonagh et al. (2005) found that while CBT had a positive impact on abused women's PTSD symptoms, the dropout rate was 41.1%, while Swift and Greenberg (2014) reported dropout rates as high as 28.5% for CBT treatment groups in eight different comparison trials. Such high dropout raises concern regarding the utility of the approach, with 59% of psychologists surveyed believing that the exposure component was likely to increase patients’ wish to terminate treatment early (Zayfert et al., 2005). These findings suggest high dropout rates may be a key factor in practitioners’ decisions not to select CBT as the first‐line treatment for PTSD sufferers, opting instead for alternative approaches such as PDT.

Although research exploring the underutilisation of exposure‐based therapy in clinical practice are dominated by US‐based studies (Becker et al., 2004), studies from the Netherlands (van Minnen et al., 2010) and Germany (Külz et al., 2010) report similar findings. In the UK, Walker and Turner (2016) raise particular concerns regarding underutilisation of exposure‐based techniques for PTSD and highlight how ‘therapist drift’ can undermine therapeutic effectiveness.

Psychodynamic conceptualisations of PTSD generally focus on the adequate formulation of a traumatised self and the associated collapse of structures, seeking to have good emerge from bad when dealing with traumatic events (Brom, Kleber & Defares, 1989; Laufer, 1988). PTSD is a normal adaptive process to an abnormal event and understanding this can lead to greater acceptance of the patient's situation and symptoms (Laufer, 1988). PDT seeks to help the client confront the traumatising event and recognise what specific circumstances mean for their life and wellbeing (Abbas & Macfie, 2013; Krupnick, 2002). Critically, psychodynamic interventions with PTSD are characterised by attempts to attain meaning in relation both to the original trauma and associated behaviours, cognitions, and affect (Kellett & Beail, 1997; Lindy, 1993). Unlike PDT, CBT attempts to account for the development and maintenance of PTSD symptoms and views re‐experiencing and arousal as conditioned responses resulting from classical conditioning during the traumatic event; the focus of CBT is not necessarily on the trauma itself but on the maladaptive behaviour that developed in the aftermath of the trauma (Foa, Keane, Friedman & Cohen, 2009).

Although studies examining PDT for the treatment of PTSD are limited, (Ponniah & Hollon, 2009), some have demonstrated its effectiveness. Using a short‐term 12‐session psychodynamic treatment approach targeting symptoms of PTSD in combat veterans, Hendin (2014) found that treatment successfully reduced symptoms of PTSD and suicidal behaviours. A review by Ponniah and Hollon (2009) found one randomised control trial (RCT) (Brom et al., 1989) comparing PDT to a control condition; patients treated with PDT reported a significant decrease in PTSD symptoms compared to a control group and a waitlist control condition. Continued improvement after treatment ends has also been reported for PDT, suggesting it may help address crucial areas in clinical presentation of PTSD and the sequelae of trauma not currently targeted by empirically supported treatments (Schottenbauer et al., 2008).

However, the results of nontrauma‐focused alternatives to CBT are inconsistent suggesting insufficient research is available to draw robust conclusions (Bradley et al., 2005; Ulrich et al., 2016). PDT has been studied less frequently than CBT and has not been studied with sufficient rigour to determine its effectiveness (Ehlers, Bisson, et al., 2010). Despite both clinicians and patients seemingly favouring PDT over CBT— Markowitz et al. (2015) reported 50% of patients preferred a psychodynamic orientated treatment and only 26% preferred CBT—few RCTs have included psychodynamic treatments for PTSD (Schottenbauer et al., 2008), leading to a lack of available evidence supporting their effectiveness and prompting questions regarding the selection of psychodynamic approaches over CBT for the treatment of PTSD.

In the light of the suggestion that, despite CBT being the recommended first‐line therapy for PTSD, practitioners are more likely to treat cases using PDT, the present review attempts to establish both the absolute and relative effectiveness of the two approaches. The aim is to further inform treatment decisions, providing insight into the treatment efficacy of psychodynamic approaches and address concerns regarding CBT for the treatment of PTSD.

## Method

### Design

A qualitative review was conducted according to systematic principles for searching, screening and data extraction.

### Search strategy

A comprehensive systematic search used the multidisciplinary and subject‐specific bibliographic databases Web of Science, Psychinfo, and Science Direct to identify papers reporting studies examining the effectiveness of psychodynamic and cognitive behavioural therapies in the treatment of PTSD. Search terms are presented in Table 1. Papers were included or excluded based on the eligibility criteria detailed in Table 2.

**Table 1:** Search terms

Psychotherapy for post‐traumatic stress disorder OR PTSD
Psychoanalysis for post‐traumatic stress disorder OR PTSD
Psychodynamic therapy for post‐traumatic stress disorder OR PTSD
Cognitive behavioural therapy OR CBT for PTSD
Psychodynamic therapy versus CBT for PTSD
Effective therapies for PTSD
Exposure OR prolonged exposure versus dynamic therapy for treating PTSD symptoms
Treating symptoms of PTSD

CBT, cognitive behavioural therapy; PTSD, post‐traumatic stress disorder.

**Table 2:** Eligibility criteria formulated using the PICOS framework

	Inclusion	Exclusion
Population	Patients with a diagnosis of PTSD, all ages	Patients with a comorbid disorder
Intervention/Comparison	CBT which included exposure, psychodynamic therapy or psychodynamic/psychoanalytic‐based therapy (including psychodynamic insight‐orientated therapy, and interpersonal therapy) Includes exposure components as well as cognitive components	Cognitive programs that do not include exposure, or vice versa If comparing either CBT or psychodynamic to another form of therapy other than the aforementioned or use of drug comparison
Outcomes	Improvement or nonimprovement of PTSD symptoms. Severity of PTSD symptoms, distress, preoccupations with trauma, dissociation, intensity of PTSD symptoms, anxiety, depression, nightmares, functioning, guilt. Effectiveness of treatment is mentioned. Dropout rates are mentioned, however, not excluded if this was not mentioned	No outcome measurement, record or comment on improvement or effectiveness
Study design	RCT, follow‐up studies, case studies, all sample sizes	Meta‐analysis

CBT, cognitive behavioural therapy; PTSD, post‐traumatic stress disorder; RCT, randomised control trial.

### Eligibility criteria

Inclusion and exclusion criteria were formulated according to the Population, Intervention, Comparison, Outcome, Study design (PICOS) framework (Counsell, 1997) and are outlined in Table 2. Criteria involving age or gender were not applied as the available evidence does not suggest that these factors affect treatment outcomes for PTSD (Etkin & Wager, 2007; Jun, Zoellner & Feeny, 2013). Samples that included comorbid disorders were excluded on the basis that they may detract from PTSD as the focus of the review. Information regarding dropout rate was included as high dropout from CBT exposure therapy has been suggested as a significant factor influencing clinical practitioners’ decisions regarding the chosen course of treatment (Follette & Ruzek, 2007; Zayfert et al., 2005). All study designs were included in the review on the basis that PDT has few RCTs investigating its effectiveness.

### Screening and data extraction

**Figure 1:**
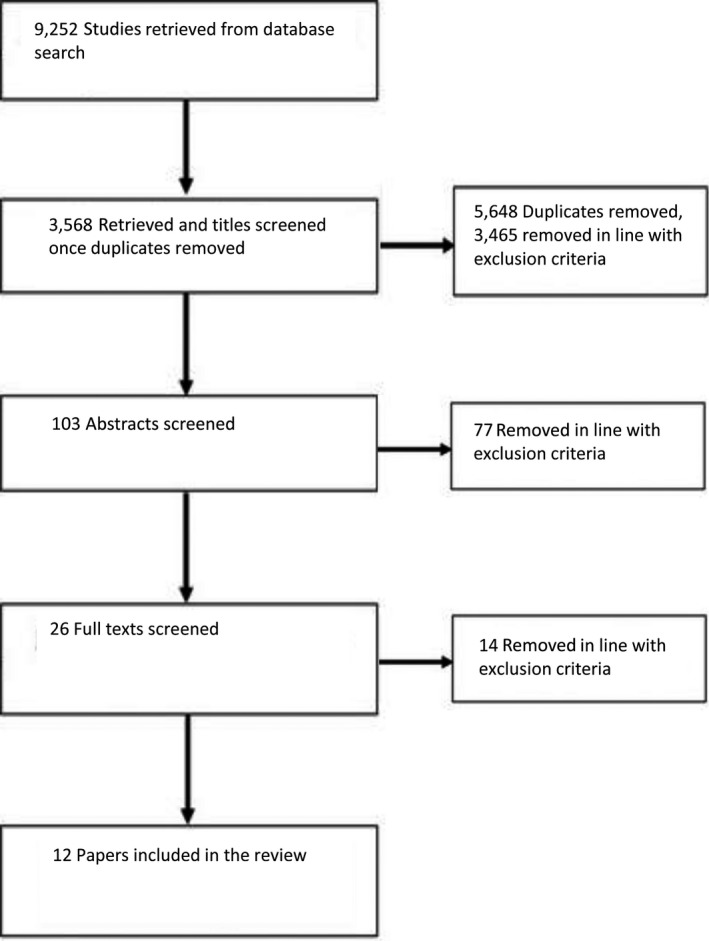
Data search and screening overview (PRISMA).

Figure 1 provides an overview of the review process and paper selection. The initial search identified 103 papers satisfying inclusion criteria; all went forward for title and abstract screening and 77 papers were removed. A total of 26 studies were subject to full‐text screening, with 12 studies judged to have met the inclusion criteria and subsequently included for full review. Author(s), population, study design, intervention(s), dropout, outcomes measure(s), results, and follow‐up data for the 12 papers selected for review were extracted into data tables summarised in Table 3. Assessment of methodological quality was guided by CASP (Critical Appraisal Skills Programme) critical appraisal checklists and is presented in Table 3 with additional narrative synthesis across subsequent sections of the review.

**Table 3:** Studies included for full review

Author(s)	Design	Intervention	Sample size	Dropout	Results	Maintenance and follow‐up
D'Andrea and Poole (2012)	Naturalistic	CBT (PE) versus TF‐PDT	*N *=* *27	Not discussed	No significant change in symptoms from either intervention. PDT associated with improvements more so than CBT	Not discussed
Levi et al. (2015)	Comparative effectiveness	CBT (PE) versus PDT	*n* (CBT) = 148 *n* (PDT) = 95	Similar dropouts reported for each intervention	Significant symptom reduction for CBT and PDT; no significant difference between treatment interventions	Significant symptom reduction maintained at follow‐up for both interventions; no significant differences found between treatment groups at any of the assessment points, including 8–12‐month follow‐up
Gilboa‐Schechtman et al. (2010)	RCT	CBT (PE) versus PDT (time limited)	*N *=* *38	Identical dropout rates of 21% for both interventions	CBT participants reported larger symptom reduction than PDT participants; CBT with exposure was superior, although both were successful in reducing symptoms	Results maintained at follow‐up. Both CBT and PDT successful in reducing distress at 6 and 17‐month follow‐up
Nacasch et al. (2011)	RCT	CBT (PE) versus PDT as treatment as usual (TAU)	*N *=* *30	CBT = 2 PDT as TAU = 2	Post‐treatment symptom severity was significantly lower in patients receiving CBT	Significant reduction in severity maintained at follow‐up for CBT but not TAU
Sijbrandij et al. (2007)	RCT	CBT (CPT) versus waitlist control	*n* (CBT) = 79 *n* (control) = 64	Not discussed	CBT group showed significant reduction in PTSD, anxiety and depression scores at 1 week post‐treatment compared to waitlist controls	No significant differences between CBT and waitlist control groups at 4‐month follow‐up
Markowitz et al. (2015)	RCT	CBT (PE) versus interpersonal psychotherapy	*n* (CBT) = 38 *n* (IPT) = 40	IPT = 10% CBT = 5.6%	Significant and comparable pre–post‐treatment symptom improvement in CBT and IPT groups but CBT group showed more rapid improvement	Not discussed
Lampe et al. (2014)	Naturalistic follow‐up	TF‐PDT	*N *=* *43	58%	Significant improvements in PTSD symptoms, global symptom load, and depressive symptoms	Significant reduction in symptoms (60% in depression, 74% in PTSD symptoms, and 76% in global symptom load) at 2‐year follow‐up
Britvić et al. (2006)	Prospective cohort study	PDT (long‐term group)	*N *=* *59	17 of 59 (28.8%)	Significant reduction in intensity of PTSD symptoms; no change in neurotic symptoms or defence mechanisms	Not discussed
Kellett and Beail (1997)	Case study	Psychodynamic interpersonal psychotherapy	*N *=* *1	Not discussed	Rapid decrease in symptoms.	Reductions in symptomology maintained at follow‐up
Monson et al. (2006)	RCT	CBT (CPT) versus waitlist control	*n* (CBT) = 30 *n* (control) = 30	CBT = 16.6% control = 13%	CBT group showed significant post‐treatment reduction in symptom severity; 40% did not meet PTSD criteria and 50% had reliable change in PTSD symptoms at post‐treatment assessment	30% of CBT and 3% of waitlist controls did not meet criteria for PTSD at 1‐month follow‐up
Hinton et al. (2004)	RCT	CBT (CPT) versus control	*n* (CBT) = 6 *n* (control) = 6	Not discussed	CBT showed significant improvement with large effect sizes	Not discussed
Abbas and Macfie (2013)	Case study	Supportive and insight‐oriented psychodynamic psychotherapy	*N *=* *1	Not discussed	Significant improvement in all from the pretreatment baseline phase to the total treatment phase	Patient contacted therapist twice in a 6‐month period and reported continued effective functioning

CBT, cognitive behavioural therapy; PTSD, post‐traumatic stress disorder; RCT, randomised control trial.

### Clinical diagnostic

Each study included for full review reported conducting formal diagnosis procedures to confirm PTSD in the sample. Britvić, Radelić and Urlić (2006), Lampe, Barbist, Gast, Reddemann and Schüßler (2014), Monson et al. (2006), Nacasch, Fostick and Zohar (2011), Sijbrandij et al. (2007), and Gilboa‐Schechtman et al. (2010) used the PTSD Symptom Scale – Interview Version (Foa, Riggs, Dancu & Rothbaum, 1993). Britvić et al. (2006) and Monson et al. (2006) also used the Clinician Administered PTSD Scale (CAPS) (Blake et al., 1995), as did Levi, Bar‐Haim, Kreiss and Fruchter (2015), and Markowitz et al. (2015). D'Andrea and Poole (2012) used the Brief Symptom Inventory and Dissociative Experiences Scale, while Kellett and Beail (1997) used a structured clinical interview based on the DSM‐IV criteria.

## Results

Studies involved participant samples from the USA (four), Israel (three), the UK (one), Vietnam (one), the Netherlands (one), Germany (one) and Croatia (one). All studies were based on adult samples with the exception of Gilboa‐Schechtman et al. (2010), which used an adolescent sample aged 12–18 years.

Three studies found PDT was an effective form of treatment for PTSD. Using an insight‐orientated psychodynamic intervention treatment, Abbas and Macfie (2013) tracked PTSD symptoms over 29 months, reporting significant phase effects from pretreatment baseline to total treatment phase for overall distress (*r *=* *−.42, *p *=* *.002), preoccupation with trauma (*r *=* *−.73, *p < *.001), and dissociation (*r *=* *−.86, *p < *.001). Kellett and Beail (1997) employed a psychodynamic interpersonal psychotherapy treatment approach, with nightmares, degree of fear, degree of upset and recovery from nightmares as outcome measures. Findings revealed a steady decline in symptoms related to frequency and distress, with no reported nightmares at night at 6‐month follow‐up. Using trauma‐focused inpatient PDT, Lampe et al. (2014) reported symptom reduction of 60% on the Beck Depression Inventory (Beck, Steer & Carbin, 1988), 74% on the Inventory of Life Changing Events (Siegrist & Geyer, 1983) and 76% on the Symptom Checklist‐90‐R (Franke, 2002) at 2‐year follow‐up compared to admission status, although there were no significant improvements in dissociative symptoms.

Despite the positive outcomes reported by Abbas and Macfie (2013), Kellett and Beail (1997), and Lampe et al. (2014), not all the studies involving psychodynamic‐based approaches yielded universally significant positive results. Britvić et al. (2006) measured symptoms of PTSD intensity, neurotic symptoms, and defence mechanisms in a study of long‐term dynamic orientated group psychotherapy. Whilst results gathered using the Clinician Administered PTSD Scale (Blake et al., 1995) showed reduced intensity of PTSD symptoms following treatment, neurotic symptoms and defence mechanisms showed no significant change, even after up to five years of treatment. In addition, evidence provided from control groups in longitudinal treatment intervention studies suggests that PTSD has a variable course, dependent on factors including population, traumatic event, and community context, but with a trend towards reduced symptomology, intensity and prevalence over time (Santiago et al., 2013). The absence of control groups in the studies conducted by Abbas and Macfie (2013), Kellett and Beail (1997), Lampe et al. (2014), and Britvić et al. (2006), limits the extent to which improvements in outcomes can confidently be attributed solely to the treatment intervention.

Only two studies directly comparing CBT and PDT, and considering their effectiveness in the treatment of PTSD, reported PDT to be equally or more effective than CBT. D'Andrea and Poole (2012) found PDT was associated with greater reductions in PTSD and depressive symptoms (*r *=* *−.47, *p *<* *.05, for both). Moderate but nonsignificant associations were also reported, with greater reductions in interpersonal sensitivity (*r *=* *−.35, *p *<* *.10), anxiety symptoms (*r *=* *−.40, *p *<* *.10), and improvement in anxiety‐related attentional biases (*r *=* *−.30, *p *<* *.10). No associations between CBT and any of the symptom measures were reported, although a moderate but nonsignificant association between prolonged exposure and worsening of anxiety‐related attentional biases (*r *=* *−.38, *p *<* *.10) was reported. Levi et al. (2015) compared CBT to PDT, focusing on frequency and intensity of symptoms, depressive symptoms, and global functioning as outcome measures. No differences were found between CBT and PDT groups in symptom levels and functioning at any assessment point and none of the interaction effects were significant, indicating equal efficacy for both treatments. Eight‐ to 12‐month follow‐up data revealed only small differences in levels of patient remission, with 33% of patients treated with PDT and 36% of patients treated with CBT remaining in remission.

Other studies directly comparing CBT to PDT reported CBT as more effective in the treatment of PTSD. Gilboa‐Schechtman et al. (2010) reported that CBT led to a larger mean reduction in PTSD symptom scores compared to PDT, 19.4 and 10.8, respectively, with pre‐ to post‐treatment effect sizes of *d *=* *1.71 and *d *=* *0.87 (Cohen, 1988), again, respectively. For depressive symptoms, those in the CBT condition using prolonged exposure reported greater reduction in Beck Depression Inventory scores (13.95) compared to those in the PDT condition (6.94). Scores on general functioning were significantly higher at post‐treatment (68.03) compared to pretreatment (54.53) across both conditions, with participants in the CBT prolonged exposure condition reporting a greater increase in scores (18.24) in comparison with those in the PDT condition (8.74). Nacasch et al. (2011) measured frequency and severity of PTSD, depression, anxiety, and trauma‐related thoughts and beliefs. Significant mean differences in PTSD Symptom Scale (Interview Version) scores were reported post‐treatment, with participants in the CBT group recording the lower mean score (18.6 vs. 35.3). Follow‐up data also showed significant change from pretreatment to follow‐up for CBT but not for PDT.

Markowitz et al. (2015) reported similar pre‐ to post‐treatment effect sizes for interpersonal psychotherapy (*d *=* *1.99) and CBT (*d *=* *1.69) measured using the Clinician‐Administered PTSD Scale (CAPS), and comparable remission rates of 23% and 26%, respectively, for the two treatment approaches. However, improvements were more rapid in the CBT condition, with response rates of 63% and 47%, respectively, for interpersonal psychodynamic psychotherapy and CBT patients as measured on the Clinician‐Administered PTSD Scale (Blake et al., 1995).

The remaining studies included in the review that examined CBT, but not in direct comparison to PDT, also reported CBT as an effective treatment for PTSD. Using a CBT versus delayed treatment design, Hinton et al. (2004) reported a fall from 3.3 to 1.8 on the Harvard Trauma Questionnaire (Mollica et al., 1992)—which assesses the presence of PTSD–in the treatment group compared to a fall from 3.1 to 2.0 in the delayed treatment group. Monson et al. (2006) also found that participants receiving CBT had a significant reduction in the severity of their PTSD symptoms compared to a waitlist control group. Forty per cent of CBT participants and 3% of waitlist controls did not meet any diagnostic criteria for PTSD post‐treatment. CAPS scores indicated reliable improvement post‐treatment in 50% (*n *=* *15) of CBT participants and 10% (*n *=* *3) of waitlist controls, with no reliable change in 50% (*n *=* *15) and 80% (*n *=* *24) of CBT participants and waitlist controls, respectively. Zero per cent (*n *=* *0) of CBT participants and 10% (*n *=* *3) of waitlist controls had reliably worsening symptoms. At 1‐month follow‐up, 30% (*n *=* *9) of CBT participants and 3% (*n *=* *1) of waitlist controls did not meet criteria for PTSD. Finally, while Sijbrandij et al. (2007) reported significantly lower PTSD scores (*t*(109) = 3.53, *p *=* *.001), and anxiety and depression scores (*t*(107) = 3.05, *p *=* *.003) in a CBT treatment group compared to waitlist controls at one week (*t*(109) = −3.53, *p *=* *.001), at 4‐month follow‐up, the differences were no longer significant (*t*(84)* *= −.98, *p *=* *.33; *t*(87) = .85, *p* = .40). At 4‐month follow‐up, PTSD was diagnosed in 14 (26.4%) of participants who had received CBT and 21 (43.8%) of the waitlist control group.

### Dropout rates

Some authors have identified high dropout rates as partly responsible for resistance to using CBT in clinical practice (Hans & Hiller, 2013; Kar, 2011; McDonagh et al., 2005; Zayfert et al., 2005). In support of this, five of eight CBT studies did record dropout (Gilboa‐Schechtman et al., 2010; Levi et al., 2015; Markowitz et al., 2015; Monson et al., 2006; Nacasch et al., 2011). However, studies using PDT also recorded dropout, with Britvić et al. (2006) reporting 17 (28.8%) of the 59 recruited veterans dropping out of the study. Lampe et al. (2014) reported an 88.6% (31 of 33 participants) dropout while Markowitz et al. (2015) reported greater dropout in the PDT treatment group (10%) compared to those in the CBT treatment group (5.6%). Based on this evidence, concerns regarding higher risk of dropout should not be used to justify selecting PDT over CBT for the treatment of PTSD.

## Discussion

Psychodynamic therapy was found to be effective in the treatment of PTSD in the majority of studies. Lampe et al. (2014) found significant reductions on all outcome measures associated with PTSD, with more than half the participants no longer meeting the criteria for PTSD at follow‐up. Britvić et al. (2006) found long‐term group PDT had significant positive effects on reducing symptoms of PTSD, while Kellett and Beail (1997) reported that psychodynamic interpersonal psychotherapy was effective in reducing symptoms of PTSD, in particular nightmares and depression.

While each study provides evidence supporting PDT as an effective treatment for PTSD, the absence of a control group to mitigate the effects of naturally occurring time‐dependent reductions in symptomology, intensity and prevalence in PTSD (Santiago et al., 2013) represents a fundamental design limitation. In studies comparing PDT with CBT, only one study found PDT to be as effective as CBT (Levi et al., 2015), and only one study found PDT to be more effective than CBT (D'Andrea & Poole, 2012) in the treatment of PTSD. D'Andrea and Poole (2012) suggest that high dissociation, common in PTSD, and the emotional challenge of a direct approach to trauma content inherent in exposure, may have prevented traumatised clients from engaging with affective trauma memories and explain the superior efficacy of PDT over CBT. Furthermore, D'Andrea and Poole (2012) argue that strict adherence to treatment protocol of prolonged exposure is required to achieve the expected benefits. Similarly, Levi et al. (2015) state that treatment manuals were not employed and that therapy sessions were not directly monitored, implying that it is not possible to assess the level of adherence to treatment protocol. Levi et al. (2015) also highlight that patients receiving PDT received double the sessions to those treated with CBT.

Although there is some limited support for PDT, support for the treatment efficacy of CBT is more convincing. Nacasch et al. (2011) found significantly improved scores for both CBT and PDT treatment outcomes, but improvements were more marked for those receiving CBT. Furthermore, Markowitz et al. (2015) found that both CBT and PDT produced significant improvements, but these were more marked and more rapid for CBT. Finally, Gilboa‐Schechtman et al. (2010) also noted that reductions in outcome scores and improvement in symptoms were more significant for those in the CBT treatment group.

These findings suggest the superior effectiveness of CBT over PDT for the treatment of PTSD, including greater efficacy of CBT treatment at follow‐up, despite participants in a number of studies receiving PDT for a longer period. Levi et al. (2015) argue that if a shorter evidence‐based therapy such as CBT helped as much as the longer psychodynamic treatment, it would make more sense to use the shorter treatment. However, there are reports of increased anxiety and increased distress associated with CBT treatment involving exposure therapy (Cahill et al., 2006; Follette & Ruzek, 2007; Gaston, 2015; Levi et al., 2015; van Minnen et al., 2010). Additionally, the present review only included studies of PTSD without comorbid disorders. That PTSD commonly co‐occurs with other psychiatric disorders—epidemiologic surveys indicate that the vast majority of individuals with PTSD meet criteria for at least one other psychiatric disorder, and a substantial percentage have three or more other psychiatric diagnoses (Brady, Killeen, Brewerton & Lucerini, 2000)—presents a potential limitation in the review. Equally, although each study reported diagnosing PTSD prior to commencement of the study, approaches to screening varied, including both interview and self‐report measures, which may have introduced a further limitation in terms of the comparability of samples.

Dropout from exposure components is cited as a principal reason for underutilisation of CBT in clinical practice (Follette & Ruzek, 2007; Imel, Laska, Jakcupcak & Simpson, 2013; Lilienfeld, 2007; van Minnen et al., 2010; Zayfert et al., 2005). Unsurprisingly then, high dropout rates, between 16% and 21%, were reported in most studies adopting a CBT treatment approach (Gilboa‐Schechtman et al., 2010; Levi et al., 2015; Markowitz et al., 2015; Monson et al., 2006; Nacasch et al., 2011). More surprising were reports of higher dropout rates in studies where PDT was the selected treatment approach. Lampe et al. (2014) only included results of 58% of the original sample due to dropout, with only 43 of 81 participants’ data classed as ‘therapy completers’ at follow‐up. Britvić et al. (2006) reported a final data set that included only 42 of the original sample of 59 war veterans suffering from PTSD. In studies that included both CBT and PDT treatment groups, dropout rates were either comparable (Gilboa‐Schechtman et al., 2010; Monson et al., 2006; Nacasch et al., 2011), or greater in the PDT group – 10.0% in the PDT treatment group compared with 5.5% in the CBT treatment group (Markowitz et al., 2015). That similar dropout rates were reported for PDT and CBT challenges high dropout as a justifiable reason for practitioners failing to select CBT as a first‐line treatment for PTSD.

## Conclusion

Current guidelines recommend CBT as the first–line treatment for PTSD. Yet, in practice, therapists and clinicians are more inclined to adopt PDT as an alternative treatment approach. In absolute terms, the evidence reviewed supports PDT as an effective treatment for PTSD, but in relative terms, the evidence weighs in favour of CBT as the more effective treatment approach. This leads to the conclusion that current guidelines recommending CBT as a first‐line therapy should be routinely observed in the treatment of PTSD. Concerns regarding elevated patient distress associated with exposure techniques must be considered in selecting the most suitable treatment approach, but there is no evidence to support increased dropout rates resulting from exposure in CBT treatment groups when compared with groups treated with PDT. If practitioners view the risk of dropout as greater in the case of CBT, this is a misconception that should not be used to justify selecting alternative treatment approaches. There remains a need to identify other potential barriers explaining practitioners’ resistance to CBT‐based interventions in the treatment of PTSD, such as lack of training and failure to disseminate research findings to practice. In this case, better familiarity with the different methods available to introduce exposure and varying the degree and manner in which patients confront the traumatic event during therapy, together with awareness of the evidence supporting more moderate approaches, may lead to greater utilisation of CBT in practice. Also, PTSD represents only one manifestation of trauma and the term ‘complex trauma’ now captures those symptoms not included in the current DSM‐5 criteria for diagnosis of PTSD, but still experienced by trauma victims. Previously these symptoms may have been subsumed under co‐morbidity, dual diagnosis or associated features, correctly underlining the complexity of trauma but resulting in complex diagnoses that, inevitably, influence clinical decisions and choice of treatment approaches (Australian Centre for the Study of Sexual Assault, 2014). Hence, clinical decisions regarding treatment must remain pragmatic. It is the provision of personalised care, addressing individual problems with recognition of the heterogeneity of symptoms in trauma populations and the development of treatments that promote tailoring interventions according to patient needs, that are the most likely to result in improvements in outcomes (Cloitre, 2015). Equally, such decisions should reflect current guidelines and evidenced‐based recommendations for the most effective treatment approach which, in the case of PTSD, currently focus on trauma‐focussed CBT interventions. Finally, the need for research to acknowledge ‘trends’ in practice and engage in studies further investigating the efficacy of psychodynamic approaches for the treatment of PTSD should not be overlooked.
